# Mr. Howe, on Amputation

**Published:** 1810-02

**Authors:** John Howe

**Affiliations:** Assistant-Surgeon on board H. M. ship Princess Carolina. *Yarmouth Roads*


### *To the Editors of the Medical and Physical Journal.*

GENTLEMEN,

SURGEONS, after describing amputation, as it is most commonly performed by compressing the artery with the tourniquet, mention, where this cannot be had recourse to, the next step is to remove the extremity at the joint; without mentioning those cases which occur, where amputation is required, where no tourniquet can be applied from its nearness to the trunk, and where there is sufficient room to operate without opening the joint. That these cases frequently occur I have but little doubt.

In an attack on the Russian gun-boats, near Fredericksham, on the night of the 25th of July 1809, I accompanied the boats of the squadron under Capt. Pater, which were commanded by Capt. Forest. The force of the English was upwards of three hundred, including officers and men; men; the enemy's between two and three hundred. The victory was gained on our side by the loss of 70 killed and wounded. The enemy suffered in killed and wounded, about 100. The wounded English were accommodated on board a transport brig that was captured at the same time, until they were conveyed on board their own ships. This was done early in the morning of the 26th. The greater part of our men were wounded by the enemy's musquetry. One of the men belonging to the Cerberus frigate, had the os humeri broke about its middle by a musket ball. As I considered this a case requiring amputation, so soon as I had examined it, I placed the limb in an easy posture, and accommodated him with a birth, where he could lie horizontally, until he was sent to the care of his own surgeon; which was done in the course of five or six hours after. As soon as the men were sent on board their respective ships I returned on board my own, which was the senior officer's ship; he gave orders that the wounded prisoners should be sent on board the captured brig. There was no Russian surgeon with them. Mr. Noble, surgeon (at that time) of this ship, and myself, as soon as we had accommodated our own wounded men, went on board the brig to dress their wounded, &c. In the operations which were required to be performed, Mr. Noble and myself took our turns. The last man on whom we had occasion to operate, was wounded by a musket ball in the upper part of the arm; the os humeri was splintered. Mr. Noble said, that nothing else could be done here, but to remove the arm at the shoulder joint, and asked me, as it was my turn to operate, which way I intended to perform it? I proposed tying the artery in the axilla as the first step of the operation, and observed, that I thought there was no necessity for laying open the joint, as no extraneous body had already done it. This being agreed to, I proceeded to tye the axillary artery, having taken proper care to dissect it from the brachial nerves; after which I divided the muscles with a common scalpel, one by one, and took up each artery as soon as it was divided; by this means little blood was lost: the bone was then divided with a saw close to the insertion of the capsular ligament, and the integuments were brought over to produce union by adhesion. On the 27th the wounded prisoners were sent from the brig to a house on shore. On the 3d day the wound was dressed; no discharge was found confined, and the appearance clean and healthy. This man walked about from the time of the operation, and had no symptomatic fever;

 his his spirits and appetite good. On the 6th day after the amputation he was sent to Russia; at this time the wound was adhering and looked well, with very little discharge.

Mr. Noble and myself went on shore every morning, to attend the wounded prisoners; about the 28th, the surgeon of the frigate came on shore, just as we had done dressing the wounded men; he said the case which is mentioned, belonging to the frigate, he had amputated, and not having room for the tourniquet, he compressed the artery with a key, at the clavicle, and operated below the shoulder-joint, in the usual way as when you have a tourniquet on the limb; that the man had lost much blood during the operation, and was at this time very low. One of the gun boats, on the morning of the 26th, after the action, had gone alongside the frigate, and did not send her wounded men on board the brig, which were fifteen in number, until the surgeon of the frigate had dressed their wounds, and in one case had amputated the fore-arm; in this gun boat, which was alongside of the frigate, one of the Russians was wounded in a way very similar to the case on which I operated near the shoulder-joint, excepting that there was a much larger external wound extending down the arm; I did not see this man until the 27th, when much tension and inflammation had come on. On the day the surgeon of the frigate came on shore, I proposed in this case, that fomentations and poultices should be applied, and when the inflammation had subsided, to remove the arm, which met the approbation of the surgeons. This man was sent soon after to his own country, which prevented the operation. The joint in this case had not been opened by the shot, or the suppuration which followed.

Would it not be best to avoid in every instance opening the joint, where no extraneous body has previously done it, as the cure will be so much earlier accomplished, and the treatment of so many troublesome sinuses avoided? It is not at the shoulder-joint that I conceive this mode of operating will be confined to; a much greater convenience will be found by attending to this, in cases where the hip-joint is not laid open, and the injury so high up, that you have not sufficient room to apply a tourniquet. I know not if any one has ever recovered from amputation at the hip-joint. I have little doubt that it will be admitted, that it is not a desirable operation. Should I meet with a case where a shot has struck the os femoris, and splintered it so high up as not to leave room to apply the tourniquet, and still the cavity of the joint not laid open, I would tie the femoral femoral artery a little below Poupart's ligament; cut the muscles through with a scalpel; tie the arteries the same as in operating for the removal of a large tumour, and proceed afterwards as in the common amputation.

I am, &c.

JOHN HOWE,

Assistant-Surgeon on board H. M. ship Princess Carolina, *December* 29, 1809. *Yarmouth Roads.*

